# Ciliated foregut cyst of the pancreas: Preoperative diagnosis using endoscopic ultrasound guided fine needle aspiration cytology—A case report with a review of the literature

**DOI:** 10.4103/1742-6413.56362

**Published:** 2009-10-09

**Authors:** Kulwinder S Dua, Aravind S Vijayapal, Janis Kengis, Vinod B Shidham

**Affiliations:** Division of Gastroenterology and Hepatology, Department of Medicine, Medical College of Wisconsin, Milwaukee, WI, USA; 1 Gastroenterology Specialists, SC, Waukesha, WI, USA; 2 Department of Pathology, Medical College of Wisconsin, Milwaukee, WI, USA

**Keywords:** Ciliated foregut cyst, cystic lesion, cytology, endoscopic ultrasound, fine needle aspiration, pancreas

## Abstract

A 51-year-old male presented with a 4-month history of abdominal pain, decreased appetite, and postprandial bloating. A CT scan showed a solitary, 5.3 × 4.4 cm, cystic lesion in the body/tail of the pancreas. Endoscopic retrograde cholangiopancreatography did not show communication between the pancreatic duct and the cystic lesion. Endoscopic ultrasound (EUS) examination revealed a 6.9 × 2.4 cm cystic lesion in the body/tail region of the pancreas without septae or solid components. The pancreatic parenchyma, pancreatic duct, and common bile duct were unremarkable. EUS-guided fine needle aspiration (EUS-FNA) was performed using a 22-gauge EchotipTM needle. Only a few drops of viscous fluid could be aspirated. Papanicolaou-stained direct smears and SurePath (Autocyte) preparations were evaluated. The direct smears were hypocellular; however, the concentration method producing liquid-based cytology preparation showed detached ciliary tufts (degenerated debris with ciliated cellular fragments of cell tops without nuclei) and occasional intact ciliated cells consistent with a ciliated foregut cyst. Although benign, the cyst was resected to alleviate the symptoms. The surgical pathology confirmed the benign preoperative interpretation of the ciliated foregut cyst. To the best of our knowledge, this is the first case of pancreatic ciliated foregut cyst reported to be diagnosed preoperatively by EUS-FNA. For a proper preoperative cytologic diagnosis, the needle rinses should be processed adequately. Otherwise, these hypocellular specimens with mucin may be misinterpreted as mucinous cystic lesions.

## INTRODUCTION

Endoscopic ultrasound (EUS) and EUS-guided fine needle aspiration (EUS-FNA) cytology are commonly being used for the diagnosis and staging of esophageal, gastric, rectal, pancreatic, biliary, and other tumors.[1] One of the important indications in pancreas is the evaluation of pancreatic cystic lesions. Neoplastic pancreatic cystic lesions include serous cystadenoma, mucinous cystic neoplasms, intraductal papillary mucinous neoplasms, cystic pancreatic ductal adenocarcinomas, and cystic endocrine tumors. Among the nonneoplastic cystic lesions of the pancreas, although pancreatic pseudocysts are common, other cysts in this category include retention cysts, congenital or developmental cysts, para-ampullary duodenal wall cysts, enterogenous cysts, cystic acinar transformation, endometrial cysts,[2,3] parasitic (e.g., echinococcal) cysts, and other squamous cell-lined cysts such as lymphoepithelial, epidermoid, and dermoid cysts.[4–6] Several of these neoplastic and nonneoplastic pancreatic cysts are discovered incidentally on CT/MRI scans done for other indications. The preoperative characterization of these cysts to precancerous/cancer versus benign cysts without malignant potential can be difficult and hence several patients are subjected to either surgery or to sequential follow-up scans. Here we report a case of pancreatic ciliated foregut cyst that was diagnosed preoperatively by EUS-FNA. This preoperative diagnosis avoided the need to do extensive surgery as is required for mucinous cystic lesion as per recommended algorithms.[7]

## CASE REPORT

A 51-year-old male presented with a 4-month history of intermittent right upper quadrant pain and decreased appetite without a known history of GI tract or pancreaticobiliary disease. The transient, nondebilitating, and vague pain was unrelated to oral intake or bowel movements. He also noted worsening postprandial bloating without nausea or vomiting and had lost 16 pounds over the past month due to food aversion. He consumed one to two alcoholic drinks per day and denied heavier use in the past.

**Figure 1:**
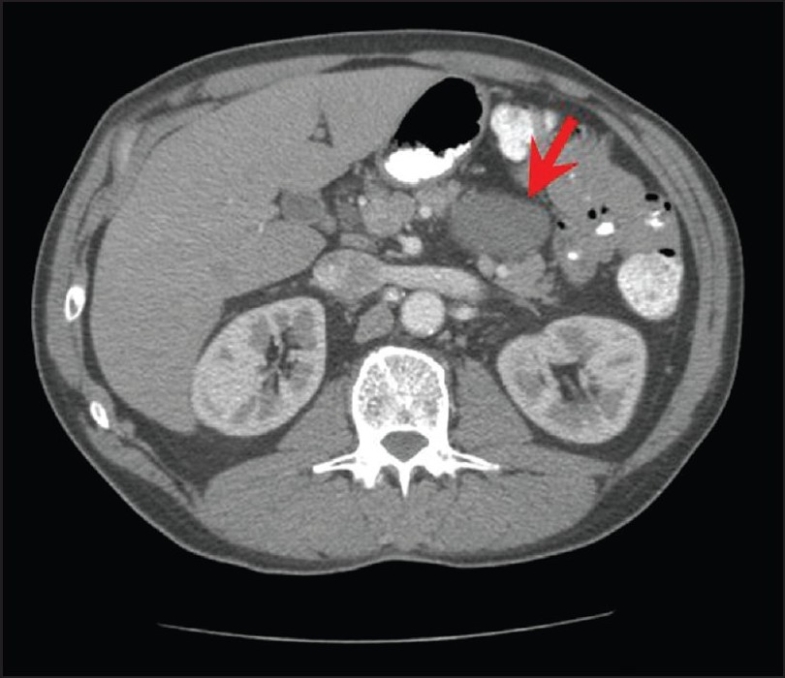
CT scan of the abdomen shows a 5.3 × 4.4 cm cystic lesion (arrow) in the body/tail of the pancreas without any changes in acute or chronic pancreatitis

A CT scan of the abdomen showed intrapancreatic, solitary, 5.3 × 4.4 cm, cystic lesion in the body/tail of the pancreas [Figure 1] with differential diagnosis of either a mucinous cystic lesion or a pseudocyst. The pancreatic parenchyma, liver, gall bladder, and biliary tree were unremarkable. Magnetic resonance cholangiopancreatography showed normal pancreatic and intra/extra-hepatic bile ducts. Endoscopic retrograde cholangiopancreatography (ERCP) did not show communication between the pancreatic duct and the cystic lesion. There was no ductal ectasia. The patient was referred for EUS.

**Figure 2:**
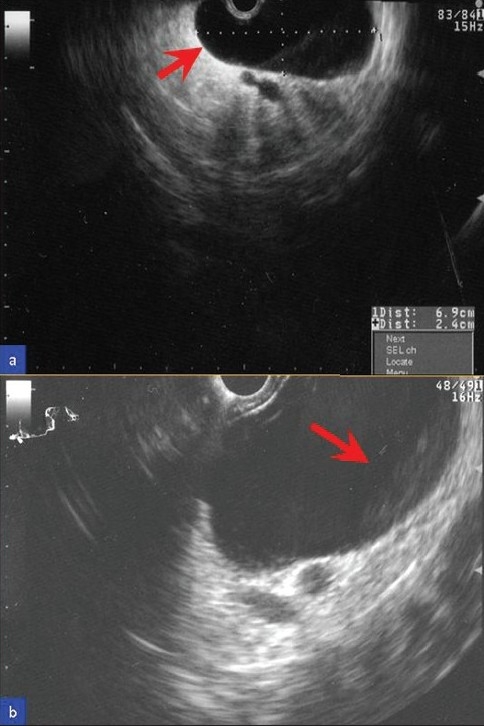
Ultrasound image of the pancreatic cystic lesion. (a) EUS showed a 6.9 × 2.4 cm cyst (arrow) in the pancreatic body–tail region without septae or solid component. (b) Under higher magnification, there is a subtle layering of debris (arrow) within the cyst

**Figure 3:**
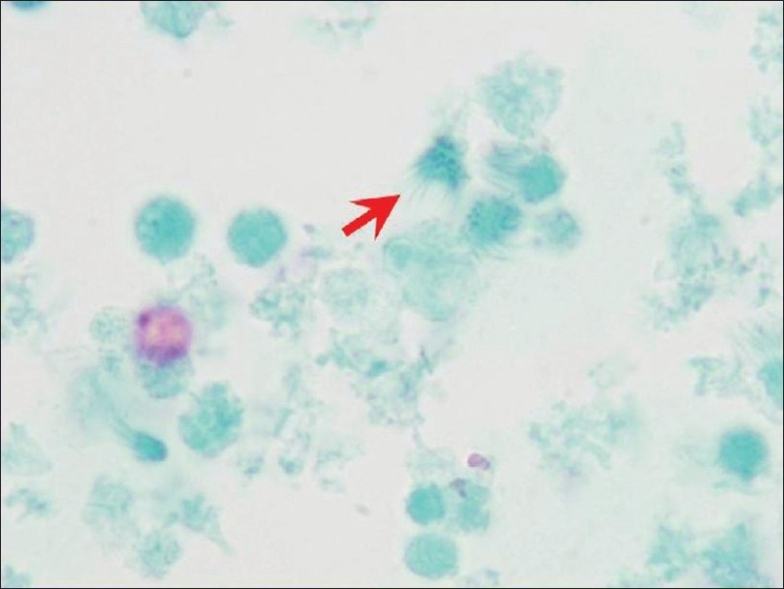
EUS-FNA cytology. The hypocellular preparation predominantly showed debris with detached ciliary tufts (red arrow). Goblet cells and keratinous debris and/or anucleated squamous cells were not present (Papanicolaou-stained SurePath preparation)

**Figure 4:**
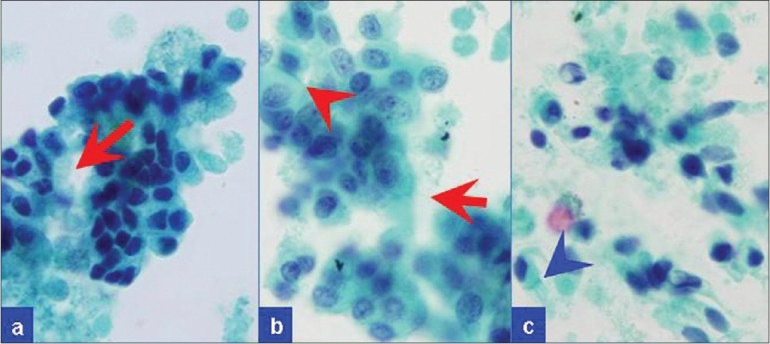
EUS-FNA cytology. Rare to find were small groups of cells with cilia (red arrows) in some areas. Terminal bars (blue arrowheads) were discernible in some isolated ciliated cells (Papanicolaou stained SurePath preparation)

**Figure 5:**
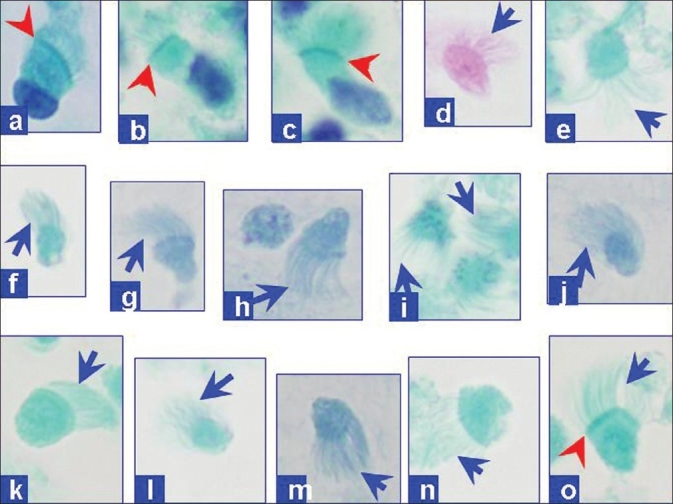
EUS-FNA cytology. Rare, isolated intact ciliated columnar cells with nuclei (a-c) were scattered amongst debris with relatively many detached ciliary tufts of top fragments of ciliated columnar cells without nuclei. (d-o) The cilia (blue arrows) were visible with distinct terminal bars (red arrowheads) in some (Papanicolaou-stained SurePath preparation)

EUS examination of the pancreas using an Olympus linear array EUS scope revealed only one, 6.9 × 2.4 cm, cystic lesion in the body/tail region without septae or solid components [Figure 2]. The pancreatic parenchyma, pancreatic duct, and common bile duct were unremarkable. The pancreatic duct was 1.7 mm in diameter (within normal limits) in the body of the pancreas. FNA was performed using a 22-gage Echotip needle (Cook Medicals, Winston-Salem, NC, USA). Due to the high viscosity of the cystic fluid, only a few drops of clear mucoid fluid could be aspirated. It was sent for cytopathologic evaluation. Due to an insufficient amount of the fluid, tumor markers, amylase, and lipase levels could not be determined. A few direct smears were prepared and the needle was rinsed in the CytoRich™ fixative for Papanicolaou-stained AutoCyte Prep™ (liquid-based cytology—LBC). The direct smears were hypocellular and insufficient for interpretation. Most of the cellularity was in LBC preparations showing predominantly degenerated debris with detached ciliary tufts (DTCs) and occasional preserved intact ciliated cells. The cytopathologic features were consistent with a ciliated foregut cyst of the pancreas [Figures 3–5] and ruled out a cystic mucinous lesion.

**Figure 6:**
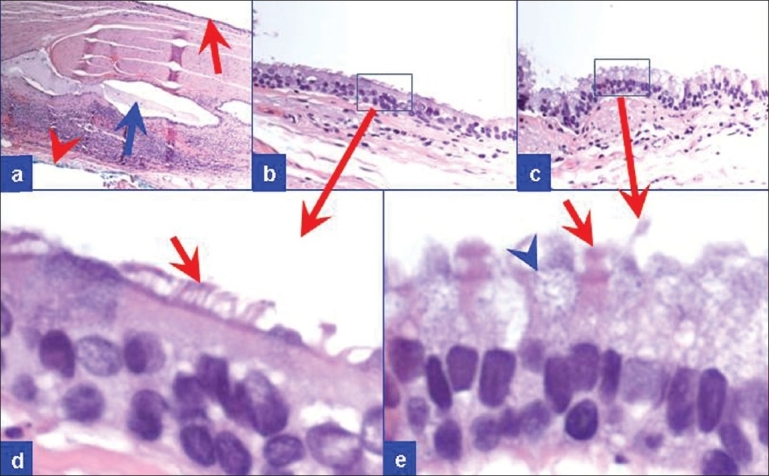
Resection of the pancreatic cyst. (a) Cyst wall with a few small cysts filled with mucin (blue arrow). (b, c) The cyst was lined by respiratory pseudostratified, tall, columnar epithelium with ciliated cells (red arrows, d and e) and interspersed with goblet cells (blue arrowhead in e) (H and E stain)

Based on symptoms of abdominal pain, food aversion, and weight loss, the patient was referred for surgical resection of this lesion. He subsequently underwent distal pancreatectomy with relief of his symptoms. The surgical pathology of the resected lesion confirmed the benign preoperative interpretation of the ciliated foregut cyst [Figure 6].

## DISCUSSION

This case highlights the significance of the preoperative diagnosis of cystic lesions of the pancreas to avoid unintended wide resection, especially in cases without symptoms. According to the recommended management algorithm, this case with a cystic pancreatic lesion otherwise may have been managed surgically by default.[7]

Congenital foregut cysts are not uncommon in the mediastinum, where they account for around 20% of all the mediastinal space-occupying lesions.[8] However, they are very rare in the peripancreatic and hepatobiliary regions. There have been fewer than 70 cases reported in the liver and an even smaller number in the peripancreatic region.[9] Congenital foregut cysts are lined by pseudostratified, ciliated epithelium and may contain mucous glands or cartilage in their walls. They may be present in the tracheobronchial tree or in the GI tract as bronchogenic or enteric cysts, respectively, and cause localized symptoms secondary to infection if the lesions communicate with the tracheobronchial tree or to physical factor such as compression.

Histologically, the wall of ciliated foregut cysts are lined by a pseudostratified, ciliated epithelium interspersed with goblet cells [Figure 6]. Rare lymphocytes may be present in the cyst wall. The ciliated epithelial cells are immunoreactive for cytokeratin (CK) 7 and CA 19-9. These cells are nonimmunoreactive for CK20, CDX-2, and TTF-1. Rare cells interspersed within the epithelial layer may show immunoreactivity for neuroendocrine markers such as chromogranin and synaptophysin.[8] The pericystic pancreatic tissue is usually unremarkable.

While these cystic lesions are benign, sporadic cases of both adenocarcinoma[10] and squamous cell carcinoma[11] have been reported. However, in general, surgical resection is not indicated unless they cause symptoms. However, these cysts are frequently misdiagnosed as pancreatic, cystic mucinous neoplasms that can lead to inappropriate surgical intervention.[12,13] There is an isolated report describing the EUS-FNA findings of a ciliated foregut cyst of pancreas evaluated retrospectively after surgicallyresection.[9] To our knowledge, this is the first case of a pancreatic ciliated foregut cyst diagnosed preoperatively by EUS-FNA.

Based on the review of the literature, there are two publications covering 11 patients on the utility of EUS-FNA cytology for ciliated foregut cysts (1 pancreas[9] and 10 mediastinal[14]). The cytologic specimens were usually hypocellular and showed degenerated cells, needle-like crystals, and amorphous debris in a mucoid/mucinous fluid background.[9] Rare, small loosely cohesive clusters of cuboidal-to-columnar cells without morphologically obvious cytoplasmic mucin may be present. One of the most consistent features reported is DCTs seen as the top portion of columnar cells without nuclear portions scattered amongst debris in relatively hypocellular specimen [Figure 5]. DCTs have also been emphasized as one of the consistent features of ciliated foregut cysts in a series reporting EUS-FNA cytology of 10 mediastinal ciliated foregut cysts.[14] A report which initially missed small number of DCTs in its case also emphasizes its significance after a retrospective review of cytologic preparations following operative resection and confirmation of the lesion as pancreatic ciliated foregut cysts.[9] A 4.5 cm, retroperitoneal bronchogenic cyst, in a 38-year-old man is reported.[12] This cyst was attached to but was separate from the pancreas. Cytological preparations of EUS-FNA showed cuboidal-to-low-columnar epithelium, and a pancreatic mucinous cystic tumor was suggested leading to surgical resection. DCTs or ciliated cells have not been mentioned by this report.[12] Cilia have also been reported in extraordinarily rare cases of ciliated adenocarcinoma of the pancreas.[15,16] However, these are not cystic lesions and the specimens should be relatively cellular with at least a moderate degree of cellular atypia.

In the current case, DCTs with rare ciliated cells facilitated the benign interpretation of the ciliated foregut cyst. The cyst content was difficult to aspirate by EUS-FNA, was hypocellular, and thickly mucoid with debris. As observed in our case, intact ciliated cells, although difficult to find, were detectable [Figures 3 and 4]. These features are mostly detectable in cytological preparations made with concentration methods such as cytospin preparations,[9] ThinPrep,[14] or SurePrep (current case). Well-preserved columnar cells are rare and difficult to find or may be absent altogether in the cytologic preparations. Occasional small, flat sheets of cells with a honeycomb pattern are reported in the background admixed with amorphous debris. These may represent a gastrointestinal mucosal contaminant. Goblet cells and keratinous debris and/or anucleated squamous cells are not present.[9,14]

Although the carcinoembryonic antigen (CEA) level in the cyst fluid could not be determined in this case (as only a few drops of the fluid could be aspirated), it may be elevated as was reported in some cases.[9,17] This finding of thick mucin may be misinterpreted as coming from a mucinous cystic lesion (mucinous cystic neoplasm or Intraductal papillary mucinous neoplasm).

There is lack of data on CEA levels in the cyst fluid to currently make any recommendation with reference to pancreatic cililary foregut cysts. A case with a ciliated hepatic foregut cyst showed elevated CA-19-9 serum levels.[18] A strong CA19-9 immunostaining of the ciliated epithelial cells of the pancreatic ciliated foregut cyst has been reported.[8] Thus to avoid inappropriate radical management, caution is warranted in interpreting CEA and CA19-9 levels of pancreatic cyst fluids.[17] This highlights the pitfall of misdiagnosing such lesions if one relies exclusively on ancillary tests such as analysis of the cyst fluid for CEA and CA19-9 or staining of smear preparations for the mucin stain in the absence of a diagnostic cellular material leading to misinterpretation as cystic mucinous lesions.

In summary, we report the first preoperatively diagnosed pancreatic ciliated foregut cyst by EUS-FNA cytology. Most of the diagnostic hypocellular material was only present in LBC preparations from needle rinses in CytoRich Red fixative. For an adequate interpretation of pancreatic cystic lesions, the needle rinses should be processed by concentration methods such as LBC preparations for detecting DCTs for the correct diagnosis of ciliated foregut cysts. The awareness about clinical resemblance of cystic mucinous lesions with ciliated foregut cysts is important to prevent management pitfalls. Preoperative diagnosis may facilitate conservative management. The current patient was symptomatic and hence opted for surgical intervention. His symptoms improved after surgery.

## COMPETING INTEREST STATEMENT BY ALL AUTHORS

No competing interest to declare by any of the authors.

## AUTHORSHIP STATEMENT BY ALL AUTHORS

Each author acknowledges that this final version was read and approved.

All authors of this article declare that we qualify for authorship as defined by ICMJE http://www.icmje.org/#author.

Each author has participated sufficiently in the work and take public responsibility for appropriate portions of the content of this article.

## ETHICS STATEMENT BY ALL AUTHORS

As this is a case report without patient identifiers, approval from *Institutional Review Board* (IRB) is not required at our institution

## EDITORIAL / PEER-REVIEW STATEMENT:

To ensure the integrity and highest quality of CytoJournal publications, the review process of this manuscript was conducted under a double blind model (authors are blinded for reviewers and reviewers are blinded for authors) through automatic online system conducted by the academic editor designated for this manuscript- **Nirag Jhala** M.D, MIAC, Professor, University of Alabama at Birmingham, Birmingham, AL 35249, USA (njhala@UAB.EDU) to complete the entire peer review process starting with assigning the reviewers to final stage of accepting/rejecting the manuscript based on reviewer input.
